# Assessing the Efficacy of Alcohol Swabbing in Preventing Bacterial Colonization of Single-Use Vials: A Pilot Study

**DOI:** 10.7759/cureus.90512

**Published:** 2025-08-19

**Authors:** George Christolias, Henry Luo, Byron Schneider, Jerry Thomas, Susan Whittier, Jaspal Singh, Allen Chen, Joseph Solberg, Reza Ehsanian

**Affiliations:** 1 Department of Physical Medicine and Rehabilitation, Columbia University Vagelos College of Physicians and Surgeons, New York, USA; 2 School of Medicine, University of New Mexico School of Medicine, Albuquerque, USA; 3 Center for Musculoskeletal Research, Vanderbilt University Medical Center, Nashville, USA; 4 Department of Rehabilitation and Regenerative Medicine, Columbia University Vagelos College of Physicians and Surgeons, New York, USA; 5 Department of Pathology and Cell Biology, Columbia University Vagelos College of Physicians and Surgeons, New York, USA; 6 Department of Rehabilitation Medicine, NewYork-Presbyterian Hospital/Weill Cornell Medical Center, New York, USA; 7 Department of Orthopaedic Surgery, University of California, Los Angeles (UCLA) Medical Center, Los Angeles, USA; 8 Department of Orthopaedics and Rehabilitation, University of New Mexico School of Medicine, Albuquerque, USA

**Keywords:** alcohol swabbing, aseptic techniques, bacterial colonization, disinfection protocols, healthcare efficiency, infection prevention, isopropyl alcohol, randomized controlled trial, single-use vials

## Abstract

Introduction

Proceduralists who use single-use vials have adopted many of the same techniques for sterilizing multi-use vials, including swabbing the rubber tops with 70% isopropyl alcohol before drawing injectate. However, the lack of literature specific to this sterilization technique for single-use vials brings into question the necessity of this practice. This pilot study aims to establish protocols and determine the feasibility of methods to assess the hypothesis that routine use of disposable alcohol swabs does not affect the risk of bacterial colonization of injectate vials.

Methods

Forty new injectate vials (n=20, vials with an aluminum cap; n=20, vials with a plastic cap) were randomly assigned to a "swab"/"no-swab" group. Vials assigned to the "swab" group were firmly swabbed once with 70% isopropyl alcohol after cap removal and allowed to air-dry. A blinded researcher sampled the exposed rubber stopper of the injectate vials by firmly swabbing the rubber with a sterile cotton culture swab dipped in sterile 0.9% normal saline. A microbiologist blinded to group assignment assessed the degree of bacterial growth from swabs of the injectate vials' rubber stoppers after incubation on blood agar plates.

Results

There was no growth in any of the 40 samples in either the "swab" or "no-swab" group on day 5 of plate incubation at 35°C in a CO2 environment. Subgroup analysis by vial cap material (plastic vs. aluminum) also revealed no detectable microbial growth in either group. Statistical testing confirmed no significant differences between groups (two-sided Fisher's exact test; P=1.00), and confidence intervals indicated a wide range of possible undetected effects due to limited power. These findings indicate that the swabbing of single-use vials prior to usage had no measurable impact on sterility under clean clinical conditions.

Conclusion

The findings of this pilot study provide valuable insights into the efficacy of routine alcohol swabbing in preventing bacterial colonization of single-use injectate vials. Despite the widespread practice of swabbing vial tops with 70% isopropyl alcohol before drawing injectate, the necessity of this protocol has been called into question due to the lack of specific literature supporting its effectiveness for single-use vials. However, our results demonstrate that neither swabbing nor the absence of swabbing resulted in bacterial growth on the rubber stoppers of injectate vials after two and five days of plate incubation. These findings suggest that for the types of vials assessed in this study, routine alcohol swabbing may not be essential for preventing bacterial contamination. Further research involving larger sample sizes and diverse types of injectate vials is warranted to confirm these findings and establish evidence-based guidelines for vial preparation protocols in clinical settings.

## Introduction

Injections from single- and multi-use vials are directed through various guidelines in the clinical setting to ensure that they minimize the risk of infection, protect patient safety, and optimize resource use [1,2]. However, while there exist clear instructions on the aseptic procedures and disinfection practices regarding multi-use vials, instructions on handling single-use vials remain unspecified, leaving room for misuse [3-5]. Both multi-use and single-use vials are methods of parenteral injection. However, multi-use vials typically contain more than one dose and may be reused given proper sterilization techniques as follows: (1) being kept and accessed in dedicated sterile zones; (2) affirming that the medication has not expired; and (3) disinfecting the top of the vial before each access. Single-use vials, however, are intended for a single dose in a single patient. Historically, inappropriate reuse of single-dose vials has led to multiple major outbreaks of infection in hospitals, affecting at least 95,000 individuals from the years 2007 to 2012 through potential exposure to infectious diseases [1,5]. Misuse of single-use vials typically involves reusing them which has well-documented guidelines from the Centers for Disease Control and Prevention (CDC), but there are no specific guidelines regarding the effectiveness or necessity of swabbing single-vial tops with an alcohol wipe (70% isopropyl) to prevent infection [2]. While the World Health Organization (WHO) broadly recommends strict hand hygiene and disinfection of any vial septa before penetration, these guidelines do not distinctly address single-dose vials. The CDC also cautions usage of alcohol as a disinfectant since it cannot penetrate protein-rich materials [6,7].

The ambiguity in guidelines raises questions about the efficacy and necessity of swabbing the tops of single-use vials with alcohol as a precautionary measure. A 1994 study regarding unnecessary disinfection procedures found incidentally that 99% of the single-dose vials tested were sterile, suggesting that further disinfection may be redundant [8]. Further review of the literature reveals many guidelines recommending cleaning the tops of any vial, including single-use vials, before use [8,9]. However, these recommendations seem to be founded more on conventional wisdom rather than on empirical evidence.

This is not the first time the need for alcohol wipes before an injection has been brought under scrutiny. There are many studies exploring the necessity of using alcohol wipes on the skin before injection. While it may seem intuitive that using an alcohol wipe to clean the site of injection will decrease the risk of infection, studies show no increase in adverse events, even in situations where there is a marked decrease in skin bacteria counts [10,11]. Studies like this reveal the unnecessary use of resources that can be better allocated elsewhere. Additionally, the WHO recommends disinfecting the entry site of an injection by wiping the skin for 30 seconds, followed by an additional 30 seconds of waiting for the alcohol to dry [7]. The recommended minute per injection may affect the efficiency of hospital procedures. Moreover, if the injection proceeds before the alcohol fully evaporates, it could cause unnecessary pain for the patient, further questioning the need for alcohol sterilization.

Given the gaps in research, the current study serves as a pilot project to establish protocols and determine the usefulness of common techniques, as well as assess the hypothesis that routine use of disposable alcohol swabs has no effect on the risk of bacterial colonization of injectate vials.

The primary endpoint of the study was the proportion of vials with any detectable colony-forming units (CFUs) on blood agar after five days of incubation. Secondary objectives were to (1) compare contamination rates between vials with aluminum caps and those with plastic caps and (2) assess the feasibility of the study protocol for potential use in larger-scale investigations with greater effect sizes.

## Materials and methods

Study design

**Figure 1:**
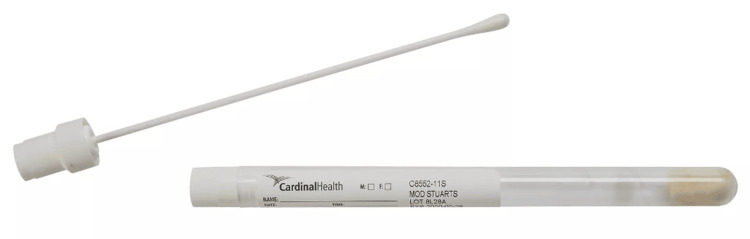
Rayon swab with a plastic shaft C8552-11 Rayon swab used to swab the tops of the vials [13]

This study utilized a double-blind, randomized controlled trial to minimize bias. Forty Hospira single-use vials, 20 10 mL 0.25% bupivacaine and 20 30 mL 1% lidocaine (20 plastic covers, 20 aluminum covers), were allocated into two groups: experimental vials which would be swabbed with 70% isopropyl alcohol using a single Rayon swab with a plastic shaft (Figure 1) and control vials which would not be swabbed with 70% isopropyl alcohol. Randomization was performed using an online randomization algorithm to ensure impartiality [12]. To simulate a clinically relevant environment, the study took place in the Harkness Pavilion at Columbia University Irving Medical Center in New York, United States, held to standard sterility guidelines with research personnel gloved and masked. 

**Figure 2:**
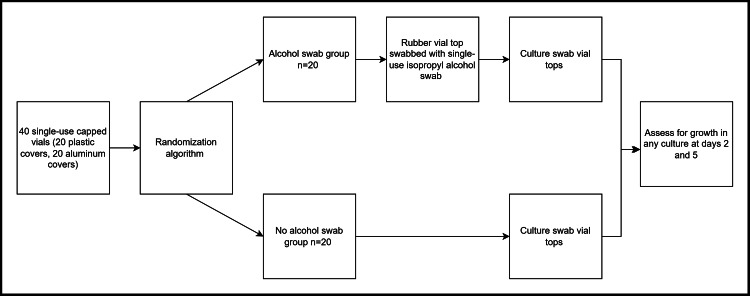
Flow diagram of the protocol

The study incorporates three general steps: (1) vial preparation, (2) sampling vials, and (3) culture assessment (Figure 2). Research personnel responsible for vial preparation and labeling were not involved in any subsequent processes, including sampling and data collection. Additionally, sampling personnel were blinded to the vial groupings, and data collection personnel were blinded to prior steps. These measures ensured that each step of the study was conducted without bias and based on pre-established criteria and protocols.

Vial preparation

Following the allocation of groups, research personnel responsible for vial preparation removed the covers on four vials and swabbed the top of the vials once using 70% isopropyl alcohol for those randomized to the experimental group. The alcohol was allowed to air-dry for a minute using a timer. Vials in the control group were not swabbed. Care was taken throughout the process to ensure there was no inadvertent contact of the research personnel with any part of the vial or cap during the cap removal or swabbing process. 

Sampling vials

Following the vial preparation, research personnel responsible for sampling used a sterile culture swab with a cotton tip that was dipped into sterile normal saline (NaCl 0.9%) to stroke the rubber of each vial firmly. This was done with the deliberate intention to avoid the aluminum sides on the top of each vial. Saline was utilized to ensure adequate retrieval of bacteria as per the protocol of the Microbiology Department of Columbia University. After retrieving the sample with the cotton tip, the swab was carefully placed into the sterile tube and assigned to its respective vial label. Multiple research personnel validated this assignment process to mitigate any human error. This step was repeated 10 times to sample all 40 vials.

Culture assessment

The samples were transferred to a Columbia University Microbiology Laboratory where they were plated on 5% sheep blood agar, chocolate agar, and MacConkey agar to cover most bacteria of clinical importance including most gram negatives, gram positives, and fastidious organisms. Bacterial cultures were chosen instead of fungal or viral assessment since they are the most common contaminant in medication vials and more efficient to culture. Research personnel responsible for culture assessment evaluated and recorded the quantity of CFU on each blood agar plate on day 5. 

Ethics statement

This project involved the in vitro testing of sealed medication vials only; no living human or animal subjects or identifiable data were used. Institutional review board approval and informed consent were not required.

## Results

Of the 40 vials that were randomly assigned to either an alcohol swab group or a control group, day 5 assessment of CFUs revealed no bacterial or yeast growth on the blood agar plates for either group (Table 1).

**Table 1:** Culture results at day 5 CFU: colony-forming unit

	Growth (CFU>0)	No growth (CFU=0)	Total
Swab	0	20	20
No-swab	0	20	20
Total	0	40	40

Group differences were assessed with a two-tailed Fisher's exact test. All 40 vials (20 swabbed, 20 unswabbed) showed 0/40 positive cultures, displaying no difference between groups (P=1.00). Exact binomial 95% confidence intervals (CI) (Clopper-Pearson) was 0-16.8% in each arm; the risk difference 95% CI was −16.8% to 16.8%. With 20 vials per arm, the study had only 80% power to detect an absolute difference of ≥34%; smaller effects could not be ruled out.

Subgroup analysis was assessed between aluminum-capped and plastic-capped vial culture results (Table 2). Both aluminum-capped (0/20) and plastic-capped (0/20) vials showed no growth; subgroup two-tailed Fisher's exact test showed no difference between groups (P=1.00) and a risk difference 95% CI of −16.8% to 16.8% in all comparisons.

**Table 2:** Culture results at day 5 stratified by vial-capped material CFU: colony-forming unit

	Growth (CFU>0)	No growth (CFU=0)	Total
Aluminum-capped vials	0	20	20
Plastic-capped vials	0	20	20
Total	0	40	40

Analyses were performed in R (4.3.2) (R Foundation for Statistical Computing, Vienna, Austria, https://www.R-project.org/) using the exact 2x2 package (v1.6.9) [14,15]. There were no missing samples or protocol deviations.

## Discussion

The results of the double-blind, randomized controlled trial revealed no growth of CFUs on the blood agar plates for either the experimental group, which had the vial tops swabbed with 70% isopropyl alcohol, or the control group, which was not swabbed. These findings indicate that the practice of swabbing the tops of single-use vials with alcohol does not significantly affect the sterility of the vials; additionally, subgroup analysis reveals that the material of the vial cap (aluminum vs. plastic) did not measurably change the sterility of the vial at least in terms of preventing bacterial growth as measured by this methodology.

Interpretation of results

The lack of bacterial growth in both experimental and control groups may propose that the additional step of swabbing single-use vial tops with alcohol may not be necessary for maintaining sterility. This is in line with the premise that single-use vials, designed for a single entry and to be discarded thereafter, maintain a high level of sterility from manufacturing to the point of use. Our findings add empirical evidence to the debate on the necessity of disinfecting single-use vials before puncture, suggesting that the risk of contamination might be inherently low or that existing aseptic techniques are sufficient to prevent contamination without the need for additional alcohol swabbing. The WHO recommends that after wiping the diaphragm of an injection vial with 70% isopropyl alcohol or ethanol, allow for the alcohol to evaporate before inserting any device into the bottle [7]. While the exact timing is not specified, even the minimum 10 seconds for *Staphylococcus aureus* and *Streptococcus pyogenes* to be killed by isopropyl alcohol would add up quickly over multiple injections [6]. It is important to note, however, that the most concerning cause of vial contamination in nosocomial settings is not *S. aureus* and *S.* *pyogenes.* 

Existing literature and guidelines

The findings of this pilot study contribute to a growing body of literature and guidelines questioning the necessity of routine disinfection practices that may not improve outcomes in low-risk clinical scenarios. Our results, demonstrating zero bacterial growth regardless of alcohol swabbing, are consistent with prior investigations on both vial disinfection and skin preparation.

Buckley et al. were among the earliest to question the necessity of swabbing single-dose vial septa, finding that 99% of unopened vials were sterile, with contamination likely arising from breaches in aseptic technique rather than intrinsic vial sterility [8]. The present study supports this conclusion by showing no bacterial growth even when vial tops were not swabbed under controlled clean-clinic conditions. Current WHO guidelines broadly recommend alcohol swabbing of vial septa prior to access [7]. However, these recommendations are extrapolated largely from best practices for multi-dose vials, where repeated access increases the risk of contamination as well as the general intuition that alcohol swabs keep "things" clean. Our study adds to the argument that for immediate application of single-use vials, disinfection protocols may be redundant when standard aseptic handling is maintained. The CDC even recommends against sterilizing medical and surgical equipment with alcohol given its weak sporicidal properties [6]. Additional general considerations include the tendency for alcohol to damage various rubber and plastic tools, as well as the need to store alcohol in a cold, well-ventilated area due to its flammability.

Limitations and error

The methodology utilized in this study presents various avenues for error. The sampling method, while designed to avoid aluminum sides and focus on the rubber septum, may not fully capture potential contaminants if they were present on the vial but not on the sampled area. Additionally, the environment in which the vials were prepared and sampled was controlled for sterility, which might not accurately reflect conditions in all clinical settings where contamination risks could be higher. Furthermore, inclusion of positive controls would help determine if the experimental setup is capable of giving positive results, as ours did not grow any cultures. 

Several limitations should be considered when interpreting the results of this study. Firstly, our sample size of 40 vials, though sufficient for a pilot study, is relatively small and will likely not capture the variability present in broader clinical practice. Specifically, the lack of power in the study means that the results do not necessarily disprove that there is a decrease in culture growth between the swabbing and no-swabbing groups. Secondly, we focused solely on bacterial growth and did not assess for other pathogens such as viruses or fungi, which could have different responses to alcohol swabbing. Thirdly, the controlled environment does not perfectly mimic all conditions under which vials are used in real-world settings, potentially limiting the applicability of our findings. Finally, Hospira supplied all vials in the study, mostly from a select few batches. Vials in the same batch are likely to have similar microbiology results, which may introduce confounding factors. Testing with vials from different suppliers could show different results. 

## Conclusions

The primary resources needed for a larger study, such as single-use vials, alcohol swabs, sterile culture swabs, and blood agar plates, are relatively inexpensive and widely available in clinical and laboratory settings. In addition, the study has minimal risks yet has direct and important clinical implications. Findings revealed no statistically significant difference between the swabbed and non-swabbed vials, indicating that swabbing with alcohol did not affect bacterial culture growth. While the true predictive value of the study remains undetermined due to its low sample size and power, it challenges the assumption that additional disinfection of single-use vials enhances sterility. Not only is using a disposable alcohol wipe potentially unlikely to improve sterility; it may, in fact, decrease efficiency in providing injections, in both time and cost, and also potentially add unnecessary pain from injection into unevaporated alcohol. Further research is necessary to test these conclusions in various clinical settings. This study aims to build an extensive understanding of the risks and benefits of several scenarios concerning swabbing single-use vials, with the intention of maximizing patient safety and resource efficiency. 

The scope of the study was limited to bacteria in vitro, as well as in size and power. In consideration of these limitations, the next steps would be to replicate the study on larger scales while also testing results for other pathogens, such as viruses or fungi. Should future in vitro studies show similar results, sterilizing single-use vials may not be recommended for best clinical practice.
